# Suspected Transmission of Severe Fever with Thrombocytopenia Syndrome Virus from a Cat to a Veterinarian by a Single Contact: A Case Report

**DOI:** 10.3390/v14020223

**Published:** 2022-01-24

**Authors:** Atsushi Miyauchi, Ken-Ei Sada, Hirotaka Yamamoto, Hiroki Iriyoshi, Yuji Touyama, Daisuke Hashimoto, Shigeru Nojima, Shingo Yamanaka, Keita Ishijima, Ken Maeda, Masafumi Kawamura

**Affiliations:** 1 Department of Internal Medicine, Kochi Prefectural Hata-Kenmin Hospital, Sukumo 788-0785, Japan; miya_atsushi_0723@yahoo.co.jp (A.M.); hr413055@gmail.com (H.Y.); hikk5296@gmail.com (H.I.); y.touyama1219@gmail.com (Y.T.); hashimotod0108@gmail.com (D.H.); sgrnjm.1965@gmail.com (S.N.); a84367870926@gmail.com (S.Y.); kawamura.m@gmail.com (M.K.); 2 Department of Clinical Epidemiology, Kochi Medical School, Kochi University, Nankoku 780-8072, Japan; 3 Department of Veterinary Science, National Institute of Infectious Diseases, Shinjuku-ku, Tokyo 162-8640, Japan; keishi@nih.go.jp (K.I.); kmeda@nih.go.jp (K.M.)

**Keywords:** severe fever with thrombocytopenia syndrome, cat-to-human transmission, human, cats, polymerase chain reaction

## Abstract

A 67-year-old male veterinarian presented with fatigue, anorexia, and diarrhea. Although there were no tick bite marks, we suspected severe fever with thrombocytopenia syndrome (SFTS) due to bicytopenia, mild disturbance of consciousness, and a history of outdoor activities. Thus, we started immunoglobulin therapy immediately. A serum reverse transcription-polymerase chain reaction (RT-PCR) test for SFTS virus (SFTSV) was positive. The patient had treated a cat with thrombocytopenia 10 days prior to admission. The cat’s serum SFTSV RT-PCR test result was positive, and the whole genome sequences of the patient’s and cat’s SFTSV were identical, suggesting the possibility of transmission from the cat to the patient. Other cases of direct cat-to-human SFTV transmission have been reported recently. Mucous membranes should be protected, including eye protection, in addition to standard precautions, when in contact with any cat with suspected SFTS.

## 1. Introduction

Severe fever with thrombocytopenia syndrome (SFTS) is a tick-borne viral infection caused by SFTS virus (SFTSV) of the genus *Dabie bandavirus*. The case fatality rate of SFTS is 10−27% in East-Asian countries such as China, South Korea, and Japan [1,2,3]. Epidemiological studies have shown that a history of breeding cats or bovines is a risk factor for infection [4], and that direct infection from cats is possible [5,6]. Herein, we report a case of SFTS in a veterinarian who treated a cat with SFTS.

## 2. Case Report

A 67-year-old male veterinarian was admitted to our hospital with fatigue, anorexia, and diarrhea. He reported mowing the grass 4 days prior to admission. On admission, his level of consciousness was 10 (E3V3M4) on the Glasgow Coma Scale. A physical examination revealed blood pressure of 144/72 mmHg; a pulse rate of 85 beats/min; body temperature of 37.2 °C; and a respiratory rate of 24 breaths/min. There were no abnormal findings in the examination of the head, neck, chest, or abdomen, and no tick bite marks or skin rash were observed. Laboratory tests revealed leukopenia, thrombocytopenia, an increased serum ferritin level, and normal CRP levels (Table 1).

We suspected SFTS due to the bicytopenia, mild disturbance of consciousness, and history of outdoor activities, and started immunoglobulin therapy immediately. Hemophagocytosis was found in a bone marrow specimen, so steroid pulse therapy was initiated on day 2. On day 4, a serum real-time reverse transcription-polymerase chain reaction (real-time RT-PCR) test for SFTSV was confirmed as positive. The patient’s level of consciousness began to improve after a few days. His ferritin level peaked on day 6 and the platelet count returned to normal by day 13. He was discharged on day 17.

During the admission, the patient revealed that he had treated a cat with SFTSV infection 10 days before admission. During the medical and mortuary care of the cat, he had worn gloves and a face mask, but no eye protection. The cat had tested positive for SFTSV on a serum real-time RT-PCR test. Whole genome sequences of SFTSV in sera from the cat and the patient were determined according to the previous report [7], revealing that both SFTSVs were completely identical (DDBJ accession number LC663817-LC663822). Thus, we concluded that the virus had been transmitted directly from the cat to the patient.

## 3. Discussion

We experienced a case of SFTS in a veterinarian with the virus transmitted directly from a cat that he had treated.

The main mode of transmission of SFTSV to humans is via SFTSV-carrying tick bites [8]. However, a recent epidemiological study revealed cat or cattle ownership as a risk factor for SFTS [4], and some cases of directly transmitted SFTSV infection from cats to humans have been reported in Japan [5,6]. Our patient treated a cat with SFTS 10 days before hospital admission, which is consistent with the incubation period of SFTS [9]. The 100% identity of the whole genome sequences of the cat’s and the patient’s SFTSV suggest that direct transmission of SFTSV from the cat to the patient may have occurred.

Transmission from SFTSV-infected cats to humans could be caused by droplet infection. Although in a previously reported case of a veterinarian with SFTS transmitted from cats, the patient had relatively intensive contact with several sick cats, our patient only had contact with a single cat and had no intensive contact. In the previously reported case, SFTSV was detected not only in the serum, but also in eye swabs, saliva, and urine of cats experimentally infected with SFTSV, and the amount of virus was particularly high in eye swabs [10]. It has been reported that SFTS antibody titers of veterinarians and veterinary nurses are higher than those of the general population [11]. Previous reports suggest that even brief contact with cats infected with SFTSV may result in SFTS transmission. A recent systematic review about the prevention of severe acute respiratory syndrome coronavirus 2 transmission found that eye protection, in addition to use of a face mask, might be effective in preventing direct viral transmission [12]. The presence of the same SFTSV in the patient and the cat suggests transmission between the cat and the patient, but tick-borne transmission cannot be completely ruled out.

In conclusion, not only the tick-borne route, but also the possibility of direct animal-to-human transmission should be considered in the diagnosis of SFTS. When having contact with cats suspected of having SFTS, mucous membranes should be protected, including eye protection, in addition to standard precautions such as the use of face masks, gloves, and aprons.

## Figures and Tables

**Table 1:** The patient’s laboratory test results on admission.

Parameter	Value	Reference Range
WBC (cells/L)	1430	4100–9300
RBC (×104 cells/L)	423	430–570
Hb (g/dL)	13.6	13.7–17.5
PLT (×104/L)	6.4	13–35
AST (IU/L)	35	12–33
ALT (IU/L)	24	5–35
ALP (IU/L)	67	38–113
LDH (IU/L)	233	124–222
CK (U/L)	250	56–244
TP (g/dL)	6.8	6.7–8.3
Alb (g/dL)	4.2	3.9–4.9
BUN (mg/dL)	15.5	8–20
Cr (mg/dL)	0.80	0.61–1.04
Na (mmol/L)	129	135–147
K (mmol/L)	3.4	3.3–4.8
Ca (mg/dL)	8.1	8.8–10.1
Ferritin (ng/mL)	565	30–310
CRP (mg/dL)	0.34	0–0.3

Alb, albumin; ALP, alkaline phosphatase; ALT, alanine aminotransferase; AST, aspartate aminotransferase; BUN, blood urea nitrogen; CK, creatine kinase; Cr, creatinine; CRP, C-reactive protein; Hb, hemoglobin; K, potassium; LDH, lactate dehydrogenase; Na, sodium; PLT, platelets; RBC, red blood cells; TP, total protein; WBC, white blood cells.

## Data Availability

The data presented in this study are available on request from the corresponding author. The data are not publicly available due to the patient’s privacy.
